# Laparoscopic Clipping of the Inferior Mesenteric Artery for the Treatment of Type II Endoleak Using Intra-operative Indocyanine Green Fluorescence Angiography

**DOI:** 10.1016/j.ejvsvf.2026.06.005

**Published:** 2026-06-25

**Authors:** Irene D. Kikkert, Steven J.G. Leeuwerke, Harry G.M. Vaassen

**Affiliations:** a Department of Vascular Surgery, Medisch Spectrum Twente, Enschede, The Netherlands; b Multimodality Medical Imaging Group, TechMed Centre, University of Twente, Enschede, The Netherlands; c Fluorescence Imaging Lab, Medisch Spectrum Twente, Enschede, The Netherlands

**Keywords:** Endoleak, Endovascular aneurysm repair, Indocyanine green fluorescence, Inferior mesenteric artery, Laparoscopy

## Abstract

**Introduction:**

Type II endoleaks (T2ELs) are a common finding after endovascular aortic repair (EVAR), often caused by retrograde flow from the inferior mesenteric artery (IMA). Although transarterial embolisation is standard, laparoscopic IMA ligation may provide a definitive alternative in selected cases. Indocyanine green fluorescence angiography (ICG-FA) adds real time vascular visualisation, helping the identification and confirmation of successful ligation. This report illustrates the potential benefit of Indocyanine green guided laparoscopic IMA clipping for managing T2ELs.

**Technique:**

The case of a 69 year old man with an abdominal aortic aneurysm who underwent emergency EVAR for a ruptured left iliac artery aneurysm is presented. At two year follow up, computed tomography angiography revealed a persistent T2EL originating from the IMA, resulting in abdominal aortic aneurysm growth. Given the aneurysm’s progression, laparoscopic IMA clipping using ICG-FA was performed. The IMA was identified, and ICG-FA was used to confirm its precise location before applying ligation clips to occlude the vessel. Successful exclusion was further confirmed with repeated ICG-FA. Post-operative recovery was uneventful, and no endoleaks were detected on follow up imaging (computed tomography angiography).

**Discussion:**

ICG-FA laparoscopic clipping of the IMA may offer a safe and effective treatment option in selected cases for persistent T2ELs after EVAR. Compared with endovascular options, it allows direct exclusion of the inflow vessel with real time visual confirmation. ICG-FA can provide real time functional imaging of vascular perfusion, enabling intra-operative identification of the IMA and objective confirmation of successful vessel occlusion. Its integration is practical, widely accessible, and may increase the safety and confidence of laparoscopic IMA ligation in selected patients.

## Highlights

• Laparoscopic clipping of inferior mesenteric artery (IMA) treats type II endoleaks.
• Indocyanine green fluorescence angiography (ICG-FA) may aid IMA identification.
• ICG-FA enables real time confirmation of successful IMA vessel occlusion.
• ICG-FA is widely available and may be easily integrated into standard laparoscopy systems.

## Introduction

Endovascular aneurysm repair (EVAR) has become the preferred treatment for abdominal aortic aneurysms (AAA) due to reduced peri-operative mortality and morbidity rates, and shorter hospital stays compared with open repair. However, re-interventions remain common, occurring in up to 25% of patients within five years after surgery.^1^ Specifically, type II endoleaks (T2ELs) are a common complication after EVAR, reported in up to 22% of patients.^2^ Most T2ELs stem from retrograde flow from the inferior mesenteric artery (IMA). Although most do not require treatment, persistent T2ELs can lead to aneurysm growth and an increased risk of rupture and therefore require re-intervention.^2^^,^^3^

Transarterial embolisation, typically using coils or embolic agents (e.g., ethylene vinyl alcohol copolymer such as Onyx), remains the most commonly reported initial treatment strategy for T2ELs.^4^ However, technical success varies widely, with persistent or recurrent endoleaks reported in up to 72% of cases.^5^ Laparoscopic clipping of the IMA has emerged as an effective and increasingly adopted alternative for patients who are suitable for surgery.^6^ This approach allows direct exclusion of the inflow vessel. However, identifying and isolating the IMA can be technically challenging, particularly in patients with previous abdominal interventions.

Indocyanine green fluorescence angiography (ICG-FA) enables real time visualisation of vascular perfusion and may support rapid intra-operative vessel identification and confirmation of vessel occlusion. Despite its potential benefits, the reported application of ICG-FA in this context remains limited to reported case studies.^7^^,^^8^ In this video, the authors illustrate laparoscopic IMA clipping with adjunctive ICG-FA, specifically highlighting its potential role in supporting intra-operative vessel identification and confirmation of successful occlusion.

## Technique

A 69 year old man with a history of hypertriglyceridaemia, type II diabetes mellitus, and a conservatively managed acute coronary syndrome presented with acute abdominal pain. Computed tomography angiography (CTA) revealed a ruptured left common iliac artery aneurysm (CIAA; 79 × 79 mm anteroposterior diameter [APD] × transverse diameter [TD]) with a retroperitoneal haematoma. Additionally, bilateral internal iliac artery aneurysms and an AAA of 49 × 49 mm (APD × TD) were identified, which had not been diagnosed previously.

The patient underwent emergency EVAR with an Anaconda stent graft (Anaconda AAA Stent Graft System, Vascutek Ltd, Inchinnan, UK). An intra-operative T1bEL originating from the right limb was detected and successfully treated with additional ballooning of the affected segment, as shown on the completion imaging (Supplementary Video S1).

Supplementary video related to this article can be found at https://doi.org/10.1016/j.ejvsvf.2026.06.005

The following is/are the supplementary data related to this article:

Video S1

At the three month follow up, duplex ultrasound (DUS) and subsequent CTA demonstrated a T2EL originating from the IMA with stable AAA diameters. After two years, DUS and subsequent CTA revealed significant shrinkage of the CIAA and internal iliac artery aneurysms, a sign of successful treatment,^9^ but showed expansion of the AAA to 50 × 58 mm (ADP × TD) mm, representing a 9 mm (TD) increase in maximum diameter over a two year period. No T1EL or T3EL or lumbar contribution was observed. CTA demonstrated contrast opacification of the aneurysm sac in direct continuity with the IMA in both the arterial and venous phase (Supplementary Video S1), supporting the IMA as the most likely source of the persistent T2EL. The IMA had a normal, slender appearance.

Given the presence of an isolated, persistent T2EL with near threshold aneurysm sac expansion, treatment was considered appropriate in accordance with current guideline recommendations.^3^ The case was discussed in a multidisciplinary team meeting. Several treatment strategies were considered, including endovascular embolisation and surgical ligation. Considering the patient’s comorbidities, anatomic considerations, and personal preferences, laparoscopic IMA clip ligation with ICG-FA was selected as the most appropriate treatment strategy. ICG-FA was used as an adjunct to enhance intra-operative certainty regarding vessel identification and to confirm complete occlusion after clipping.

The procedure was performed by an experienced colorectal surgeon, with a vascular surgeon present during the operation. Under general anaesthesia, a 10 mm optical port was introduced in the peri-umbilical region to establish pneumoperitoneum, followed by two additional 5 mm working ports under direct visualisation. The mesosigmoid and AAA were identified. The peritoneum was opened in a cranial direction. The ligament of Treitz was incised to improve exposure of the infrarenal aorta and the IMA origin.

At this stage, the suspected IMA was identified based on its anatomic position as the most caudal ventral branch of the abdominal aorta, consistent with the CTA findings. ICG-FA was used to support accurate IMA identification before clipping. Five milligrams of ICG (Indocyanine green, Fresenius Kabi AG, Bad Homburg, Germany) was administered intravenously and flushed with 10 mL of saline under near infrared fluorescence using the Rubina system (Karl Storz, Tuttlingen, Germany). The origin of the IMA from the infrarenal aorta was identified (Supplementary Video S1) and carefully isolated. Two ligating clips (Hem-o-lok PurplePlus Large Polymer Clip Ligation System; Teleflex Medical, Morrisville, NC, USA) were applied close to the origin of the IMA to securely occlude the vessel, as shown in the intra-operative footage (Supplementary Video S1). The artery was not divided. Before clip placement, ICG-FA demonstrated pulsatile IMA perfusion. After clip application, no further pulsatility or fluorescence was observed, consistent with effective occlusion. Haemostasis was achieved, and the trocars and instruments were removed. The post-operative course was uncomplicated, and the patient was discharged the next day.

At six months of follow up, DUS demonstrated a reduction in both the AAA and CIAA diameters, with no symptoms or evidence of an endoleak. The patient is currently scheduled for routine annual DUS surveillance.

## Discussion

This case illustrates the potential benefit of ICG-FA as an adjunct during laparoscopic clipping of the IMA to treat a T2EL following EVAR. Laparoscopic IMA ligation is a recognised treatment option in selected patients, as reported in the literature.^6^ The present case supports these findings and illustrates how adjunctive ICG-FA may enhance intra-operative certainty and provide real time confirmation of successful vessel occlusion.

Although a transperitoneal approach was used in this case, a left retrocolic approach is also commonly used in laparoscopic aortic surgery, providing access to the IMA and lumbar arteries. Importantly, IMA ligation can be performed without ICG-FA in experienced hands. However, compared with standard laparoscopic IMA ligation, which relies primarily on anatomic landmarks without direct feedback on vessel perfusion, ICG-FA provides real time functional imaging that may increase intra-operative certainty during both vessel identification and clip placement. In the present case, fluorescence imaging aided in intra-operative decision making by supporting confirmation of the target vessel and successful occlusion after clipping. Identification of the IMA remained primarily dependent on anatomic landmarks and careful dissection, consistent with standard practice, because ICG-FA confirms perfusion rather than vessel identity. However, in cases with more complex or altered anatomy, such as previous abdominal surgery, obesity, or challenging exposure, ICG-FA may assist in the identification and isolation of the target vessel by enhancing visualisation of perfused vascular structures in real time. Although such adjunctive confirmation may not be required in every case, it may increase intra-operative certainty in selected situations. This was particularly relevant in the present case, because the vessel was clipped but not divided. This highlights its potential role as a practical adjunct to standard laparoscopic IMA ligation.^6^

Despite these favourable results, laparoscopic IMA ligation is infrequently the preferred method for treating T2EL. This may partly reflect the fact that most published series originate from centres with established laparoscopic expertise, which may limit broader applicability.^6^^,^^7^ This approach is best suited to centres with access to surgeons with laparoscopic experience, such as colorectal or gastrointestinal surgeons, working in conjunction with vascular specialists. The present centre also has extensive experience with ICG fluorescence imaging across different surgical domains, which supported the implementation of this technique in the present case. The use of ICG-FA was not related to a lack of technical expertise but was intentionally employed as an adjunct to enhance intra-operative visualisation and confirmation of complete vessel occlusion. Because most modern laparoscopic platforms are equipped for ICG imaging and the dye itself is inexpensive and widely available, its integration into standard laparoscopic procedures is both practical and cost effective. A single 25 mg flask of ICG costs approximately €85, and because of its low cost and non-toxic profile, ICG-FA is considered an accessible intra-operative tool.^10^

A limitation of this case is that the IMA was identified as the most likely source of the T2EL based on imaging findings, rather than direct intra-operative confirmation of retrograde flow. However, CTA demonstrated clear contrast opacification of the aneurysm sac in continuity with the IMA, without evidence of lumbar contribution. In addition, follow up imaging after IMA clipping showed no residual endoleak, further supporting this interpretation. Furthermore, this report represents a single case, and current evidence on the use of ICG-FA in this setting remains limited to small case series and reports.^7^^,^^8^ As such, no conclusions can be drawn regarding its superiority over standard laparoscopic IMA ligation performed by experienced surgeons without adjunctive imaging.

Regarding colonic perfusion, some authors recommend evaluating baseline colon perfusion before IMA ligation.^7^^,^^8^ However, based on experience from open aortic repair, ligation or occlusion of the IMA is generally well tolerated, and clinically significant colonic ischaemia remains a rare complication. This is consistent with the open aortic repair literature, where routine adjunctive assessment techniques such as stump pressure measurement are not commonly performed.^11^ In the context of EVAR with a T2EL, the IMA receives only retrograde flow from collateral vessels because the endograft has already excluded antegrade flow. Clipping the IMA at its origin should therefore not compromise colonic perfusion. However, anatomic variation should be considered, as a proximally originating left colic artery may be at risk during clip placement, potentially affecting colonic blood supply. ICG-FA provides direct intra-operative visualisation of bowel perfusion and could therefore serve as a useful adjunct in selected patients with suspected compromised collateral circulation. However, no single modality is completely sensitive or specific, and endoscopic assessment may also be considered depending on the clinical context.

In summary, laparoscopic IMA clipping is a recognised minimally invasive option for the treatment of T2ELs. In the present case, adjunctive ICG-FA provided real time functional imaging of vascular perfusion, supported intra-operative identification of the target vessel and enabled objective confirmation of successful occlusion, thereby increasing intra-operative certainty. Given its low cost, wide availability, and ease of integration into standard laparoscopic systems, ICG-FA represents a practical adjunct that may enhance the safety and reliability of this established technique. Written informed consent was obtained from the patient for both the procedure and publication of the associated video and clinical details.

## Conflict of interest

None.
